# The Polish Version of the Short Scale Measuring Health Literacy in Adolescence

**DOI:** 10.34763/devperiodmed.20192303.190197

**Published:** 2019-10-27

**Authors:** Joanna Mazur, Agnieszka Małkowska-Szkutnik, Leena Paakkari, Olli Paakkari, Dorota Zawadzka

**Affiliations:** 1 Department of Child and Adolescent Health, Institute of Mother and Child, Warsaw, Poland; 2 Department of Biomedical Foundations of Development and Sexology, Faculty of Education,Warsaw University, Warsaw Poland; 3 Faculty of Sport and Health Sciences University of Jyväskylä, Jyväskylä Finland; 4 Institute of Psychology, The Maria Grzegorzewska University, Warsaw, Poland

**Keywords:** health literacy, health locus of control, measurement, psychometric analysis, adolescents, kompetencje zdrowotne, umiejscowienie kontroli zdrowia, pomiar, analiza psychometryczna, młodzież

## Abstract

**Background:**

Recent years saw the development of international tools for measuring health competencies understood as health literacy (HL). One of them is the short index Health Literacy for School–Aged Children (HLSAC) implemented by the members of the Health Behaviour in School–aged Children (HBSC) network. So far, when researching the properties of this instrument, less attention has been devoted to the correlation of HLSAC with other tools related to health.

**The aim:**

of the paper is to present selected psychometric features of the HLSAC index in a sample of Polish pupils, supplemented by the preliminary analysis of its association with the health locus of control.

**Material and methods:**

The information collected relates to 630 junior secondary school students surveyed in Poland in 2016 as part of the HLSAC questionnaire pilot study. The Multidimensional Health Locus of Control (MHLC) scale by K. Wallston was used as the additional module for Poland. It differentiates among three dimensions of health control: internal, external – dependent on other people, and external – dependent on random factors. It was checked which HLC dimension correlates most strongly with HL.

**Results:**

It was demonstrated that the HLSAC scale consisting of 10 questions has a very good reliability in the Polish version (Cronbach alfa = 0.851) and a one–factor structure. Confirmatory factor analysis supported a unidimensional model (RMSEA = 0.082; CFI=0.922; TLI=0.877). The HLSAC most strongly correlates with the internal health locus of control (r=0.376; p<0.001), slightly less with the influence of other people (r=0.153; p<0.001), while the correlation with the random factor health locus of control is insignificant (p=0.947). It was found that there is a weak, however significant, positive correlation between family affluence and HLSAC (p=0.041).

**Conclusions:**

The analyses conducted demonstrated that the Polish version of HLSAC has good psychometric features. The relatively higher correlation between HLSAC and internal rather than external health locus of control was confirmed. The practical effect of the pilot study was to develop an improved version of the HLSAC scale, which has been recommended for the mainstream HBSC 2018 survey.

## Streszczenie

**Wprowadzenie:**

W ostatnich latach powstało wiele międzynarodowych narzędzi do pomiaru kompetencji zdrowotnych definiowanych jako health literacy (HL). Jednym z nich jest krótki indeks Health Literacy for School–Aged Children (HLSAC) wdrażany przez członków sieci badawczej Health Behaviour in School–aged Children (HBSC) do badań młodzieży szkolnej. Badając własności wdrażanego narzędzia badawczego, mniej uwagi poświęcano do tej pory korelacjom z innymi instrumentami powiązanymi ze stosunkiem do własnego zdrowia.

**Celem:**

opracowania jest prezentacja wybranych własności psychometrycznych skali HLSAC w pilota--żowej próbie polskich nastolatków, wzbogacona o wstępną analizę związku kompetencji zdrowotnych z umiejscowieniem kontroli zdrowia.

**Materiał i metody:**

Dane zebrane zostały w 2016 roku w grupie 630 uczniów gimnazjum w ramach udziału polskiego zespołu HBSC w międzynarodowym badaniu wstępnym kwestionariusza HLSAC. Poza protokołem międzynarodowym uwzględniono w badaniu polskim skalę The Multidimensional Health Locus of Control (MHLC) autorstwa K. Wallstona, która uwzględnia trzy komponenty kontroli zdrowia: wewnętrzną, zewnętrzną zależną od innych osób i zewnętrzną zależną od czynników losowych. Sprawdzono, który komponent najsilniej koreluje z kompetencjami zdrowotnymi.

**Wyniki:**

Wykazano, że składający się z 10 stwierdzeń krótki indeks HLSAC charakteryzuje się w polskiej adaptacji bardzo dobrą rzetelnością (alfa Cronbacha = 0,851) i ma strukturę jednoczynnikową. Potwierdzono to konfirmacyjną analizą czynnikową, uzyskując model o dobrych wskaźnikach dopasowania (RMSEA = 0,082; CFI=0,922; TLI=0,877). HLSAC najsilniej korelował z wewnętrzną kontrolą zdrowia (r=0,376; p<0,001), nieco słabiej, ale nadal w sposób istotny statystycznie z kontrolą zależną od innych osób (r=0,153; p<0,001). Tylko korelacja z przypadkowym umiejscowieniem kontroli zdrowia okazała się nieistotna statystycznie (p=0,947). Wykazano też istotny związek kompetencji zdrowotnych z poziomem zamożności rodziny (p=0,041).

**Wnioski:**

Przeprowadzone analizy wykazały dobre własności polskiej wersji krótkiego indeksu HLSAC. Potwierdzono, że HLSAC w większym stopniu wiąże się z wewnętrzną niż zewnętrzną kontrolą zdrowia. Efektem praktycznym badania pilotażowego uzyskanie poprawionej wersji skali HLSAC, która zarekomendowana została do późniejszych badań HBSC 2018.

## Introduction

The growing complexity of societies and the demands people face when taking care of their own health, that of others, and protecting the environment, have raised the challenges for the health literacy of the population [1]. Health literacy (HL) empowers individual citizens and enables their engagement in collective health promotion [2]. As a concept it has been defined in multiple ways: varying from basic literacy skills [3] to broader competencies aiming „to gain access to, understand and use information in ways which promote and maintain good health” [4]. Particularly in the field of public health and health promotion it should be understood in multiple aspects besides the abilities directly related to reading, writing and counting. However, general (functional) literacy still provides the foundation for health literacy, which in turn is understood as a mediator among individual determinants and one’s health.

An example of an initiative with a wider international scope is the implementation of the short HLSAC index into the *Health Behaviour in School–aged Children* (HBSC) study protocol. HBSC gives a unique opportunity to compare health and health behaviour across a large number of countries in Europe and North America, conducting surveys every four years in collaboration with the WHO Regional Office for Europe. HBSC focuses on a wide range of health, education, social and family measures that affect young people’s health and wellbeing [5].

The HL instrument included in the HBSC protocol was first developed in a Finnish context [6, 7] and later validated in four European countries (Belgium, Finland, Poland and Slovakia) – [8]. Finally, 10 countries decided to include that module in the final round of the HBSC studies conducted in the 2017/2018 school year (Austria, Belgium, the Czech Republic, England, Estonia, Finland, Germany, Macedonia, Poland, Slovakia). The results of the HBSC studies will provide an opportunity to compare the HL level in different countries and different systems of education. It will also be possible to examine the association of HLSAC with subjective health indicators and health behaviours of adolescents, and to build multivariate models of health determinants taking into account the HL level. The HLSAC instrument is based on a solid theoretical framework [9] and this definition and elaboration of the construct of HL steered the instrument’s development process. The tool takes into account the multidimensional nature of HL, i.e. it contains multiple items drawn from different conceptual domains (five core domains). Based on analysis, the instrument is suitable for large–scale studies and allows comparisons of subjective HL in an international context [7, 8, 9].

The earlier–mentioned studies [6, 8] focused on the assessment of psychometric characteristics of the implemented HLSAC scale. Less attention was paid to its correlation with other tools related to health. Health locus of control (HLC), which is divided into internal and external, may be considered an important element of evaluation of health–related beliefs. Research tools for measuring HLC vary by number and the definition of its detailed dimensions. However, it is assumed that persons with a strong internal sense of control are more self–confident and more strongly believe in their own capabilities. Persons with an external sense of control feel more dependent on others and often believe that their fate is determined by chance. Compared to adults, young people more often declare an external health locus of control, which is connected with their dependence on adults, and translates to making your health condition dependent on other people’s attitudes and actions.

The health locus of control should be investigated in conjunction with health literacy. People who believe that they can keep control over their health and can influence it, more often engage in pro–health behaviours and have a higher level of self–efficacy and health literacy [10]. As regards the above definition of locus of control with reference to health, it seems obvious that an internal health locus of control is conducive to health and pro–health behaviours, which has been corroborated by research on various aspects of health and health behaviours [11, 12, 13]. In the context of health locus of control, one should bear in mind that dependence on others, i.e. an external health locus of control, should also be understood as trust in doctors and other people responsible for health. It is easier for people with a higher level of external health locus of control to accept failure during treatment or other actions taken for the benefit of health [14].

## Aim of the study

The study is aimed at conducting a psychometric evaluation of the HLSAC instrument in Polish adolescents, in terms of construct validity (using confirmatory and exploratory factor analysis), and internal consistency reliability. The correlation between the HL and MHLC sum scores was tested as part of a validation study. The following research question was formulated: *Which MHLC component is most strongly correlated with HL in the population of adolescents*?

## Material and methods

### Sample

In the spring of 2016 an anonymous survey was conducted which covered 630 pupils, including 350 boys and 280 girls. They attended the first and third grade of lower secondary school (330 and 300 persons, respectively). The average age of the respondents was 14.83 years (SD=1.10). The survey was conducted in 14 schools located in three major administrative regions (Mazovia, Silesia and Pomerania). An international questionnaire, developed for the above–mentioned pilot studies, was used. A module concerning HLC was added to the questionnaire only in Poland.

### Tools

**Fig. 1:**
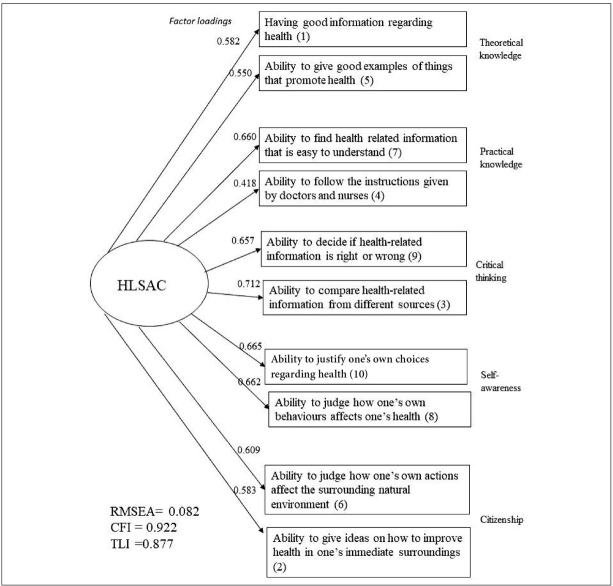
Confirmatory factor analysis model for the HLSAC index (the number in bracket corresponds to the original order of items in the questionnaire as given in the annex). Ryc. 1. Model analizy konfirmacyjnej dla indeksu HLSAC (numery pytań w nawiasie odpowiadają ich oryginalnej kolejności w kwestionariuszu, jak podano w aneksie).

The tool for the analysis of health literacy is the short HLSAC index [6] consisting of ten items. Adolescents were to respond to the statements provided on a four–level scale: *not at all true, not quite true, somewhat true, absolutely true*. The statements in HLSAC refer to five aspects of HL (theoretical knowledge, practical knowledge, critical thinking, self–awareness, citizenship), each of which is represented by two items (fig. 1). In our study an overall index ranging from 0 to 30 points was created, where the higher score represents higher health competences. In the original version, the alternative sum score ranging from 10–40 is recommended. According to the Authors’ guidelines, the levels of HL are classified into three groups depending on the coding system: “low” (score 0–15 or 10–25), “moderate” (score 16–25 or 26–35), and “high” (score 26–30 or 36–40), respectively.

Two initial translations into Polish were done from the original English version of the HLSAC. Then the translation was reconciled into one version and back– translated into English. The back translation was reviewed by the HBSC Health Literacy Group during a Skype meeting. After a pilot study, the improved version of HLSAC was included in the mainstream HBSC 2018 survey (appendix).

The second tool used was the Multidimensional Health Locus of Control Scale (MHLC) developed by Kenneth Wallston and adapted by Zygfryd Juczyński [10, 15]. The scale consists of 18 statements (assessed on a 6–level scale) with beliefs on expectations in three dimensions of the health locus of control, i.e.:

- Internality (I) – Control over my health belongs to me (internal locus of health control);
- Influence of others (O) – One’s own health status is determined by the actions of others, i.e. health care service and medical personnel (external locus of health control);
- Random (R) – Health is determined by chance or other external factors (chance or random locus of health control).

Each of the MHLC indices ranges from 0 to 30 points, and the higher the score, the stronger the belief that a given factor has an impact on health. The three levels of HLC create an individual profile of the locus of health control of a given person. Thus, a very high or a very low assessment of all levels is possible.

The following additional factors were included in the analyses:

- Demographic: gender and age;
- Socio–economic status: family affluence measured using the Family Affluence Scale (FAS), with the values from 0 to 13 points, extensively discussed in numerous publications of the HBSC research network [16]; in the sample analysed, the average FAS score was 7.63±2.36, and 9.8% of the respondents were classified into the group of definitely poor families (less than 5 points).

### Statistical analysis

Exploratory Factor Analysis (EFA) and Confirmatory Factor Analysis (CFA) were run to check the structure of the HLSAC index. In CFA the following alternative statistics was applied to evaluate the model fit: comparative fit index (CFI), Tucker–Lewis index (TLI) and root mean square error of approximation (RMSEA). According to Steiger [17], the value of RMSEA above 0.10 indicates poor fit, while CFI and TLI higher than 0.90 indicated a well–fit model [18].

Descriptive statistics and Cronbach α coefficients of internal consistency were computed for the HLSAC and MHLC sub–scales. A Cronbach’s alpha between 0.70 and 0.95 was considered good [19]. Distributions were described by mean, median and interquartile range (ICR). In order to test normality of all the frequency distributions, the Shapiro–Wilk test was applied. Spearman correlation coefficients between HLSAC score and MHLC scores were computed.

Subgroups of students were compared in terms of HLSAC and MHLC scores by means of the nonparametric Mann–Whitney test (boys vs. girls) or the Kruskal–Wallis test (family affluence groups). IBM SPSS Statistics 23 with AMOS 21 was used for statistical analysis. All the statistical analyses were two–tailed, and the results were considered significant at p<0.05.

## Results

Information about the psychometric characteristics of the scales that were used is provided in Table I. All the scales are unidimensional and have acceptable reliability. Alpha coefficients of all the scales were satisfactory, especially for HLSAC (0.851).

It has also been proven that the distribution of values of all the four scales that were analysed significantly differ from the normal curve, although their asymmetry is not substantial. The HLSAC and MHLC–I scales exhibit a slight left skewness, the MHLC–R scale – a slight right skewness, while the MHLC–O scale is the most symmetrical.

The unidimensional structure of the HLSAC index was confirmed both by the EFA and the CFA model (tab. I, fig. 1.). A one–factor solution was obtained, which explained 43.4% of the total variance in HLSAC. CFA corroborated this model with an acceptable, however not excellent goodness of fit. Standardized “path” factor loading in CFA varied from 0.428 to 0.712.

**Table I:** Psychometric properties of HLSAC and MHLC scales* (N=630). Tabela I. Własności psychometryczne skal HLSAC i MHLC* (N=630).

Scale *Skala*	Items *Pytania*	Mean ±SD *Średnia ±SD*	Median (Q1-Q3) *Mediana (Q1-Q3)*	Reliability *Rzetelność*	Factor structure EFA *Struktura czynnikowa EFA*	Shapiro-Wilk’s Test *Test Shapiro-Wilk’a*	Skeweness *Skośność*
HLSAC	10	20.66 ±4.84	21.0 (18.0-24.0)	0.851	43.4%	p<0.001	-0.414
MHLC-I	6	19.38 ±5.61	20.0 (16.0-23.0)	0.702	41.2%	p<0.001	-0.451
MHLC-O	6	14.38 ±6.67	14.0 (10.0-19.0)	0.759	45.6%	p<0.001	0.066
MHLC-R	6	13.54 ±6.07	13.0 (9.0-18.0)	0.708	41.0%	p<0.001	0.321

* all scales ranged 0-30; *wszystkie skale o zakresie 0-30 punktów*

**Annex:** Polish version of the HLSAC scale Aneks: Polska wersja skali HLSAC

	Całkowicie nieprawdziwe	Raczej nieprawdziwe	Raczej prawdziwe	Całkowicie prawdziwe
Posiadam dobre informacje na temat zdrowia	◻	◻	◻	◻
Jeśli zachodzi taka potrzeba, potrafię podać szereg sposobów poprawy zdrowia w bezpośrednim otoczeniu (np. w sąsiedztwie, u rodziny, przyjaciół)	◻	◻	◻	◻
Potrafię porównać informacje związane ze zdrowiem pochodzące z różnych źródeł	◻	◻	◻	◻
Potrafię wypełnić polecenia personelu medycznego(np. lekarza czy pielęgniarki)	◻	◻	◻	◻
Potrafię z łatwością podać przykłady działań promujących zdrowie	◻	◻	◻	◻
Potrafię określić, jak moje własne działania wpływają na otaczającą mnie przyrodę	◻	◻	◻	◻
Jeśli zachodzi taka potrzeba, wyszukuję łatwo zrozumiałe informacje związane ze zdrowiem	◻	◻	◻	◻
Potrafię określić, jak moje zachowania wpływają na moje zdrowie	◻	◻	◻	◻
Zazwyczaj potrafię się zorientować, czy jakaś informacja związana ze zdrowiem jest prawdziwa czy nie	◻	◻	◻	◻
Potrafię podać uzasadnienie moich wyborów dotyczących zdrowia	◻	◻	◻	◻

The average HLSAC index was 20.66±4.84, which means 68.9% of the maximum score (tab. I). Table II presents mean HLSAC, MHLC–I, MHLC–O and MHLC–R indices depending on gender and family affluence. Adolescents more often believe that their health is determined by themselves rather than by external factors, and mean MHLC–I indices are higher than mean MHLC–O and MHLC–R indices. Boys are significantly better than girls at assessing the health locus of control in the internal and external area which is dependent on the health care service. In the case of beliefs about the impact of other external and chance factors, the gender–related difference was small. The opposite result was found in the case of HLSAC (higher values in girls), but without statistically significant differences (p=0.087). The HLSAC index significantly improves along with an increase in family affluence. Adolescents from the poorest families more often declared that their health was determined by chance circumstances (p=0.06). Internal health locus of control exhibits the lowest economic diversity.

**Table II:** HLSAC and MHLC indices depending on gender and family affluence. Tabela II. Indeksy HLSAC i MHLC w zależności od płci i zamożności rodziny.

Scale *Skala*	Gender*Płeć*		Mann-Whitney test (p)	Family Affluence Scale (FAS) *Skala zamożności rodziny (FAS)*				Kruskal-Wallis test (p)
	Boys *Chł*.	Girls*Dz*.		very low	low	average	high	
HLSAC	20.40	20.98	0.087	19.82±4.59	20.02±4.69	20.87±5.01	21.30±4.72	0.041
MHLC-I	19.94±5.57	16.69±5.60	0.005	19.15±5.39	19.59±5.26	19.39±5.82	19.16±5.63	0.910
MHLC-O	14.99 ±6.83	13.64 ±6.39	0.013	16.20 ±6.55	14.36 ±6.26	14.31 ±6.74	13.55 ±6.88	0.113
MHLC-R	13.93 ±6.16	13.06 ±5.93	0.166	14.92 ±5.22	13.89 ±6.14	13.17 ±6.14	13.15 ±6.09	0.060

The analysis of correlations between HL and the three components of HLC found the strongest correlation with internal control (tab. III) and a nonsignificant association between HL and MHLC–R.

**Table III:** Correlation between HLSAC and three MHLC domains. Tabela III. Korelacja między HLSAC a trzema komponentami MHLC.

Component MHLC *Komponenet MHLC*	Spearman’s rho coefficient *Współczynnik korelacji Spearman’a*
MHLC-I	0.376 p<001
MHLC-O	0.153 p<0.001
MHLC-R	0.003 p=0.947

## Discussion

The paper uses the results of the pilot study conducted in Poland on a group of 630 school pupils aged 13–15 years. The focus was on examining the relationship between two scales related to health: HL and HLC, while demographic and social factors were used as the background of the analyses. The collected data have a unique value, because the MHLC scale was not included in the mainstream HBSC study. The relatively large (as for the pilot study) and geographically diverse sample increases the value of these analyses.

A significant positive correlation between HL and MHLC–I and the corresponding lack of correlation with random locus of health control demonstrates a mature attitude towards own health by the adolescents surveyed. For this reason, the correlations between the HL and internal HLC can be considered evidence of the validity of the tested tool. However, it is worth remembering that HL is a much broader concept. It refers not only to one’s own health, but also to taking care of others and the environment [7, 9]. Health literacy refers to health competencies (knowledge, skills, attitude), while health locus of control is understood as personal beliefs.

A significant association was found between family affluence and HLSAC, and a very weak one with MHLC–I, with a strong correlation between those two indices. This may mean that it is HL that is the mediator of association between family affluence and HLC. The educational trend towards strengthening the HL may thus have an impact on the appropriate formation of responsibility for one’s own health. The results obtained may be used in the future to interpret the results of studies on social inequalities in health. On the one hand, we may talk about an adverse impact of being brought up in worse conditions and with fewer possibilities to develop health competences in poorer families, while on the other hand there are worse attitudes towards one’s own health by adolescents from more neglected environments.

Differences were demonstrated in health literacy and health locus of control depending on gender. Girls had better health literacy but obtained lower scores for health locus of control, which was corroborated by the results of research carried out on a population of young adults. One explanation for those results might be that boys more often ascribe success to themselves, while girls are inclined to understand success as a joint action [20].

Health locus of control is also understood as the extent to which someone is aware that they are able to take action to promote and enhance health. Research on a population of adults has shown a correlation among health locus of control and gender, age, socioeconomic status, and stress related to economic situation, as well as social capital. The correlation between health locus of control and trust in other people and institutions is also emphasised [21, 22]. The results of research on the correlation between health locus of control and depression indicated that people with a higher level of health locus of control are less prone to depression, which means they have a higher level of self–efficacy in the context of actions taken for the benefit of health [14]. The authors of the health locus of control studies conducted among Swedish teenagers have proven that having an internal health locus of control together with the belief that health depends on powerful others significantly contributes to an increased self–esteem in adolescence [23].

This study has some limitations. The characteristics of the study participants was limited to gender, age and socioeconomic status. Additional analyses should be focused on the comparison of healthy children and chronically ill ones, as well children who differ by self–reported health. According to Berglund et al. high internal health locus of control is negatively associated with the cumulative burden of diseases, while health locus of control in chance and powerful others could be associated positively [24]. To our best knowledge, HL has not been included in this type of analysis so far.

Due to the cross sectional design of the study, the direction of the association between the variables cannot be determined. According to other authors, it can be expected that it is rather HL that affects HLC, although a mutual relation is also justified. It has also been proven that HLC mediates the relation between HL and health outcomes [25]. The collected data can help to explain the mechanism of this relation on the general (nonclinical) population of adolescents. Keeping in mind the health locus of control factor will also make it easier to interpret HBSC results.

## Conclusions

The analyses conducted demonstrated that the Polish version of HLSAC has good psychometric features. The relatively higher correlation between HLSAC and internal (rather than external) health locus of control was detected. Further research is needed to show the more complex relationship between HL, MHLC and different health outcomes. The practical effect of the research is to obtain an improved version of the HLSAC scale, which has been used in the HBSC 2018 survey (annex).
